# Effect of Regenerative Endodontic Treatment on Bone Structure in Children: A Fractal Analysis Approach

**DOI:** 10.3390/medicina61101757

**Published:** 2025-09-27

**Authors:** Ibrahim Burak Yuksel, Merve Abakli Inci, Muhammet Emin Arslan, Aysenur Cetin, Zeynep Yalcinkaya Kayhan, Kaan Orhan

**Affiliations:** 1 Department of Dentomaxillofacial Radiology, Faculty of Dentistry, Necmettin Erbakan University, Konya 42090, Türkiye; mhmmt.4295@gmail.com; 2 Department of Pediatric Dentistry, Faculty of Dentistry, Necmettin Erbakan University, Konya 42090, Türkiye; aysenur1069@gmail.com; 3 Pediatric Dentist, Private Practice, Konya 42070, Türkiye; zyalcinkay@gmail.com; 4 Department of Dentomaxillofacial Radiology, Faculty of Dentistry, Ankara University, Ankara 06500, Türkiye; call53@yahoo.com

**Keywords:** fractal analysis, radiomorphometry, regenerative endodontics, young permanent molar

## Abstract

*Background and Objectives*: This study retrospectively investigated the impact of regenerative endodontic treatments (RET) on the healing of periapical lesions in young permanent molars with open apices. Our objective was to evaluate the relationship between treatment outcomes and changes in the fractal dimension (FD) of the periapical bone before and after RET. The study was conducted at the Department of Pediatric Dentistry, Necmettin Erbakan University between January 2020 and December 2024. *Materials and Methods*: We examined panoramic radiographs from systematically healthy patients aged 6–16 years who underwent RET in the posterior mandible between January 2020 and December 2024. Changes in periapical bone were assessed using fractal analysis before treatment and after a 6-month follow-up. Additionally, mental index (MI), mandibular cortical width (MCW), mental length (ML), and periapical index (PAI) values were evaluated. Radiographs were taken with a Planmeca ProOne^®^ device and analyzed using ImageJ v1.54 software. *Results*: Comparison of FD values between treated and contralateral tooth areas, as well as before and after RET, revealed an average FD value of 1.27 ± 0.05 after regeneration, increasing to 1.29 ± 0.27 at the 6-month follow-up. Significant increases were observed in MCW (*p* = 0.005/*p* = 0.049) and ML (*p* = 0.022/*p* = 0.001) in the 35–36 and 45–46 regions post-RET, though MI values showed no significant change. Importantly, PAI scores demonstrated significant improvement after RET. *Conclusions*: The findings suggest that RET is effective in promoting the healing of periapical lesions in young permanent molars. The observed increases in cortical width and improvements in PAI scores support the positive impact of this treatment on bone healing. Furthermore, FD analysis, when combined with radiomorphometric indices, could provide a valuable and objective tool for evaluating RET outcomes.

## 1. Introduction

Managing necrotic pulps in immature permanent teeth presents a significant clinical hurdle in pediatric dentistry. The challenges primarily stem from incomplete root development and the inherently weak, fragile enamel-dentin structures characteristic of these teeth. In contrast to traditional apexification methods, regenerative endodontic treatments (RETs) have emerged as a biologically driven clinical approach aimed at restoring damaged tooth structures, including dentin, root components, and the pulp-dentin complex cells [1,2,3]. This innovative strategy is fundamentally based on principles of tissue engineering, focusing on the restoration of pulp vitality and facilitating revascularization.

Within the context of revascularization, the most straightforward and commonly employed re-scaffolding technique involves inducing an intracanal blood clot through apical bleeding. This clot effectively serves as a biological matrix, fostering the migration, adhesion, and proliferation of mesenchymal stem cells by establishing a natural fibrin network within the root canal space [4]. Crucially, several growth factors—such as platelet-derived growth factor (PDGF), vascular endothelial growth factor (VEGF), and transforming growth factor-beta (TGF-β)—released from platelets within this clot, are indispensable for promoting angiogenesis, cellular differentiation, and tissue healing processes, all vital for the successful outcome of RET [5,6]. This dynamic biological environment is particularly conducive to supporting continued root growth and apical closure in young permanent teeth [7].

Beyond clinical observations, radiographic evaluations are indispensable for assessing the success of RET. Historically, periapical and panoramic radiographs have been the primary tools used to evaluate treatment success, focusing on observable changes like apical closure and increased root length [8]. While these traditional methods provide a macroscopic view of the healing process, they often lack the sensitivity to detect subtle changes in the internal bone structure. Among these, panoramic radiography serves as a fundamental diagnostic tool in pediatric dentistry, offering a comprehensive overview of the dentoalveolar complex, including periapical regions, root development, and overall jaw structures [8]. Its broad field of view enables the assessment of multiple teeth and associated bone changes, making it particularly valuable for baseline and follow-up evaluations in cases involving immature permanent teeth. However, a limitation of these two-dimensional images is their restricted capacity to provide detailed insights into the micromorphology and intricate structural organization of bone. Consequently, advanced imaging and analytical methodologies are essential for a more precise and quantitative assessment of post-RET changes in bone structure [8,9].

Recently, advanced imaging modalities, such as fractal analysis, have gained traction for the quantitative assessment of bone trabecular architecture. Fractal analysis is a sophisticated technique that mathematically quantifies the complexity, degree of organization, and density of bone trabecular structure [9]. Specifically, the fractal dimension (FD), commonly calculated using the box-counting method, offers an objective and numerical approach to evaluate bone healing progress during regenerative processes [10]. Furthermore, this analytical method is crucial for obtaining precise and significant insights into the level of bone remodeling, particularly by accounting for inherent anatomical structures and individual anatomical variations, which can profoundly influence the healing process.

Furthermore, radiomorphometric evaluation methods, including the periapical index (PAI), mandibular cortical width (MCW), and mental index (MI), offer more than just quantitative data on bone density and cortical structural integrity. They also provide an objective reflection of the periapical region’s inflammatory regression and the advancement of bone regeneration. The PAI, a five-point scale classifying apical inflammatory lesion severity, serves as a standard tool for pre- and post-treatment comparisons. Indices like MCW and MI are valuable for assessing the structural soundness of the mandibular cortex, offering crucial information, particularly for detecting bone resorption, atrophy, or new bone formation. While these parameters can be influenced by individual variables such as age and gender, they enable the quantitative monitoring of localized morphological shifts following RET. Thus, they complement fractal analysis findings, contributing to a more comprehensive evaluation of both clinical and radiographic healing outcomes [11,12].

This study, therefore, aimed to quantitatively assess bone healing within the periapical region after regenerative endodontic treatment in young permanent molars with open apices, utilizing both FD and various established radiomorphometric indices (MCW, MI, PAI).

## 2. Materials and Methods

### 2.1. Study Design and Ethical Approval

This retrospective observational study was rigorously conducted in accordance with the principles outlined in the Declaration of Helsinki. Ethical approval was secured from the Ethics Committee for Non-Drug and Non-Medical Device Research at Necmettin Erbakan University’s Faculty of Dentistry (Approval No.: 2024/460). We strictly adhered to the STROBE guidelines for reporting observational research.

### 2.2. Sample Selection

A total of 87 patient records were initially screened based on a post hoc power analysis that verified a required sample size of 40 participants to achieve a statistical power of 0.85 to detect a medium effect size (Cohen’s d = 0.5) at a significance level of α = 0.05. A final cohort of 40 patients (15 males, 25 females) aged 6 to 14 years who had undergone RET in a permanent tooth were included in the study. These patients were treated at the Department of Pediatric Dentistry, Faculty of Dentistry, Necmettin Erbakan University, between January 2020 and December 2024. All selected patients had both preoperative and 6-month follow-up digital panoramic radiographs available.

### 2.3. Inclusion and Exclusion Criteria

**Figure 1:**
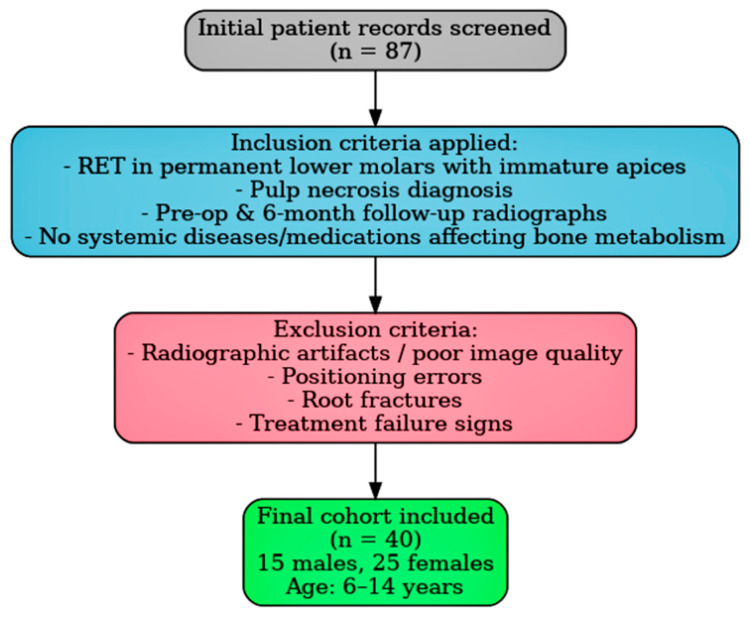
Representative panoramic radiograph demonstrating the selection of ROIs (region of interest) and the methodology for radiomorphometric measurements.

Inclusion criteria mandated that regenerative endodontic procedures were exclusively performed on permanent lower molars with immature apices and a diagnosis of pulp necrosis. Additionally, high-quality digital panoramic radiographs were available for both time points, and patients had no systemic conditions or were on medications known to affect bone metabolism. Exclusion criteria encompassed radiographic artifacts, errors in patient positioning, insufficient image quality, diagnosed root fractures, and any clinical or radiographic signs indicative of treatment failure. A detailed patient selection flowchart is presented in Figure 1.

### 2.4. Radiographic Imaging Protocol

All panoramic radiographs were consistently acquired using the same apparatus (Planmeca ProOne^®^, Planmeca Oy, Helsinki, Finland) under standardized exposure parameters (68 kVp, 7 mA, 10 s), ensuring consistency. A single, specially trained operator performed all acquisitions. Preprocessing was conducted via Romexis^®^ software (Planmeca, Helsinki, Finland) to ensure uniform brightness, contrast, and filtration across all images. Subsequently, JPEG images extracted from RAW data were utilized for all downstream analyses.

### 2.5. Image Processing and Standardization

To ensure optimal uniformity across the entire sample, all images were precisely resized to dimensions of 3840 × 1872 pixels with a resolution of 600 dpi. This process was carried out using PhotoScape X Pro (MOOII Tech, Seoul, Republic of Korea), and images were subsequently saved in TIFF format to preserve original image quality. All image analyses were conducted on a calibrated workstation equipped with an AMD Ryzen™ 5 3.2 GHz processor, 8 GB RAM, a GeForce RTX 3050 graphics card, and a 15.6-inch Full HD LCD panel (Lenovo, 1920 × 1080 pixels), operating under Windows 11 Professional.

### 2.6. Observer Calibration and Reliability Assessment

Prior to initiating data collection, a comprehensive calibration session was undertaken to ascertain intra-observer reliability. Morphometric measurements were duplicated twice, with a 24 h interval between repetitions, using panoramic images from ten individuals not included in the primary analysis. The intra-observer agreement notably surpassed 95%, as robustly demonstrated by the intraclass correlation coefficients (ICCs), thereby affirming excellent reproducibility. All subsequent image evaluations were diligently conducted by a single, blinded observer to minimize potential bias. To further ensure impartiality, standardized region of interest (ROI) selection and identical preprocessing procedures were systematically applied to every radiograph.

### 2.7. Morphometric Measurements

Before proceeding with fractal analysis, bilateral morphometric evaluations were meticulously performed at the level of the mental foramen. These evaluations encompassed measurements of MCW, mental length (ML), and the panoramic mandibular index (PMI).

MCW was precisely defined as the perpendicular thickness of the mandibular cortical bone, measured from a tangent line drawn along the inferior border of the mandible to a point intersecting the midpoint of the mental foramen. ML was represented as the vertical distance between the lower margin of the mental foramen and the inferior border of the mandible. The PMI was subsequently calculated as the ratio of MCW to ML. All measurements were performed by a single calibrated examiner, adhering to standardized image settings to guarantee consistency and minimize potential measurement error.

### 2.8. Fractal Analysis

**Figure 2:**
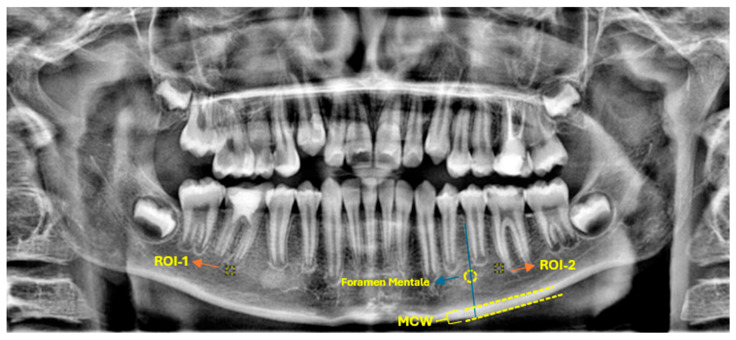
Representative panoramic radiograph demonstrating the selection of ROIs and the methodology for radiomorphometric measurements.

Fractal analysis was executed using ImageJ software (version 1.54; National Institutes of Health, Bethesda, MD, USA), which is freely accessible via the NIH website. The methodology for estimating the FD was based on the box-counting technique, as originally described by White and Rudolph. For each panoramic radiograph, a square ROI of 30 × 30 pixels was consistently selected approximately 1 mm apical to the root apex of the treated tooth (Figure 2).

**Figure 3:**
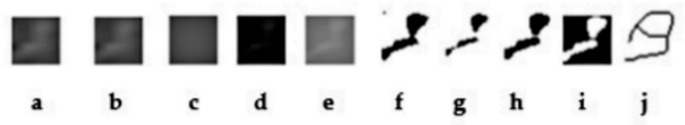
(**a**) Cropped image of the relevant area; (**b**) Duplicated image; (**c**) Application of Gaussian filter; (**d**) Image subtracted from the original; (**e**) Grayscale adjustment; (**f**) Binarization; (**g**) Erosion; (**h**) Dilation; (**i**) Inversion; (**j**) Skeletonization.

**Figure 4:**
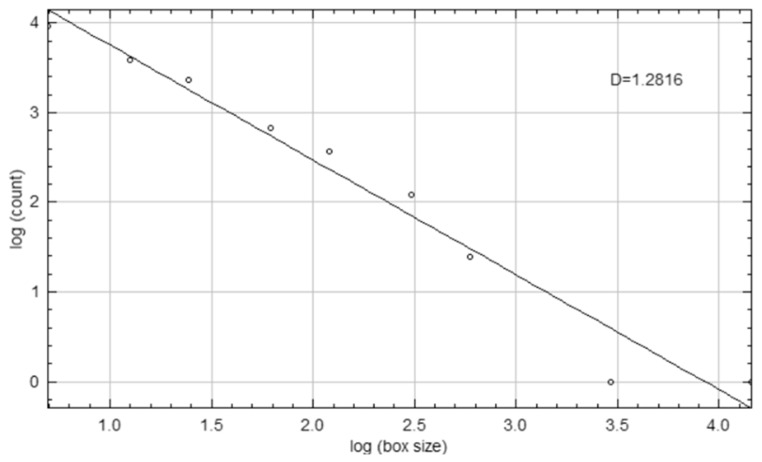
Log-log plot illustrating the calculation of the FD (D) using the box-counting method.

A corresponding ROI of equivalent dimensions was precisely positioned at the symmetrical contralateral site, serving as an internal control. ROI placement was performed with meticulous care to avoid interfering with anatomical structures, such as the mandibular canal and developing tooth germs. To further enhance consistency and precision, images were rotated and magnified as necessary before ROI selection. Each ROI was subsequently cropped and duplicated. A Gaussian blur (σ = 35 pixels) was applied to the duplicate image to suppress variations attributed to soft tissue. The blurred image was then subtracted from the original to enhance the contrast of trabecular structures, followed by the addition of a constant grayscale value (128). The resulting image was then binarized into a black-and-white format. Subsequent noise reduction was achieved through the sequential application of “erode” and “dilate” operations. The processed images were then inverted and skeletonized to enable a detailed visualization of the trabecular structure (Figure 3). FD was calculated using the “Fractal Box Count” plugin within ImageJ, employing a range of grid sizes (2, 3, 4, 6, 8, 12, 16, 32, and 64 pixels). The number of occupied boxes was plotted against box size on a double-logarithmic graph, with the slope of the regression line interpreted as the FD (Figure 4). All analyses were conducted independently for both the treated and control sites to ensure internal validity.

### 2.9. Radiographic Assessment of Periapical Status

The periapical status of each treated tooth was evaluated using the five-point PAI scoring system developed by Ørstavik et al. [13]. In this system, Score 1 indicates healthy periapical tissues, whereas Score 5 signifies severe apical periodontitis accompanied by exacerbated radiolucency. Both baseline and 6-month follow-up panoramic radiographs were independently assessed by a single calibrated examiner who was blinded to all clinical information. To ensure consistency and facilitate clinical interpretation, PAI scores were dichotomized: scores of 1 or 2 were interpreted as indicative of a healed periapical condition, while scores ranging from 3 to 5 were classified as non-healed, aligning with established criteria in the endodontic literature. Intra-observer reliability for PAI scoring was robustly confirmed on a random subset of 10 radiographs not included in the primary dataset, yielding a Cohen’s kappa coefficient of 0.91, which denotes almost perfect agreement.

### 2.10. Statistical Analysis

All statistical analyses were meticulously performed using IBM SPSS Statistics for Windows (version 22.0; IBM Corp., Armonk, NY, USA). The Shapiro–Wilk test was utilized to assess the normality of continuous data. Paired samples t-tests were employed for intra-group comparisons of pre- and post-treatment measurements (e.g., FD, morphometric indices). Independent samples t-tests were conducted to investigate gender-related differences. Cohen’s d effect sizes were calculated for changes in FD to quantify the practical significance of the observed differences. Categorical variables, including PAI-based healing outcomes, were presented as frequencies and percentages. Comparisons of categorical data between groups were analyzed using Pearson’s chi-square (χ^2^) test. A correlational analysis between age and FD values was performed using Spearman’s rank correlation coefficient (ρ) due to the non-parametric distribution of age-related data. Descriptive statistics were consistently presented as mean ± standard deviation (SD) for continuous variables and as *n* (%) for categorical variables. All statistical tests were two-tailed, and a *p*-value < 0.05 was considered statistically significant. Given the retrospective nature of this study, no a priori sample size calculation was conducted. However, as previously stated, a post hoc power analysis, focused on the primary outcome (FD change), confirmed that the final sample size provided a statistical power of 0.85 to detect a medium effect size (Cohen’s d = 0.5) at α = 0.05. This approach is considered acceptable for a retrospective study design and provides a solid foundation for the statistical validity of the findings.

## 3. Results

A total of 40 pediatric patients (mean age: 9.1 ± 2.3 years; 15 males and 25 females) who satisfied the predefined inclusion criteria were included in the analysis. All participants had undergone regenerative endodontic procedures on a permanent tooth and possessed high-quality, paired panoramic radiographs for both baseline and 6-month follow-up evaluations.

### 3.1. Fractal Dimension (FD) Outcomes

The Shapiro–Wilk test confirmed that FD values fulfilled the normality assumption (*p* > 0.05). In the treated group, the mean FD value significantly increased from 1.19 ± 0.08 at baseline to 1.27 ± 0.05 at the 6-month follow-up (*p* = 0.001). This significant enhancement indicates increased apical trabecular complexity following regenerative treatment. Conversely, a non-significant increase in FD values was observed in the contralateral control teeth, rising from 1.25 ± 0.05 to 1.29 ± 0.27 (*p* = 0.301). While this difference did not achieve statistical significance, the observed trend towards convergence of FD values between the treated and control sites suggests a progressive structural normalization in the treated regions. Table 1 provides a detailed comparison of mean FD values before and after treatment, alongside the corresponding changes in the contralateral teeth.

No statistically significant correlation was found between patient age and post-treatment FD values (r = −0.07; *p* = 0.85), indicating that the degree of trabecular complexity was independent of age. Similarly, gender-based comparisons revealed no significant difference in FD values between males (1.25 ± 0.05) and females (1.28 ± 0.05) (*p* = 0.051, independent samples *t*-test). Although females exhibited a slightly higher mean FD, this difference did not reach statistical significance. Table 2 presents the mean FD values at the 6-month follow-up, stratified by age groups and gender.

### 3.2. Mandibular Morphometric Measurements

Complementary to the fractal analysis, morphometric assessments demonstrated statistically significant increases in cortical bone thickness (MCW) and ML post-treatment: MCW increased from 1.68 ± 0.32 mm to 1.90 ± 0.33 mm on the left (*p* = 0.005) and from 1.70 ± 0.27 mm to 1.83 ± 0.34 mm on the right (*p* = 0.049). ML increased from 5.10 ± 1.22 mm to 5.72 ± 1.15 mm on the left (*p* = 0.022) and from 5.23 ± 1.25 mm to 5.77 ± 1.19 mm on the right (*p* < 0.001). In contrast, PMI values remained unchanged (left: *p* = 0.990, right: *p* = 0.371), indicating stable proportionality despite localized growth. While age-related growth is expected in pediatric populations, the consistent changes in treated regions and the absence of similar trends in contralateral controls suggest a treatment-induced remodeling effect (Table 3).

### 3.3. Radiographic Assessment of Periapical Status (PAI)

At baseline, 37 out of 40 individuals (92.5%) presented with PAI scores ≥ 3, indicative of existing apical pathology. At the 6-month follow-up, all patients demonstrated radiographic healing, with 33 individuals (82.5%) exhibiting a PAI score of 1, and the remaining 7 individuals (17.5%) showing a PAI score of 2. Importantly, no cases retained PAI scores ≥ 3. This marked improvement was statistically significant, as corroborated by the Pearson chi-square test (χ^2^(3) = 67.72, *p* < 0.001) and the Linear-by-Linear Association test (*p* < 0.001), thereby providing robust evidence for the radiographic resolution of apical lesions following regenerative therapy. Table 4 illustrates the PAI score distribution and statistical analysis results.

**Figure 5:**
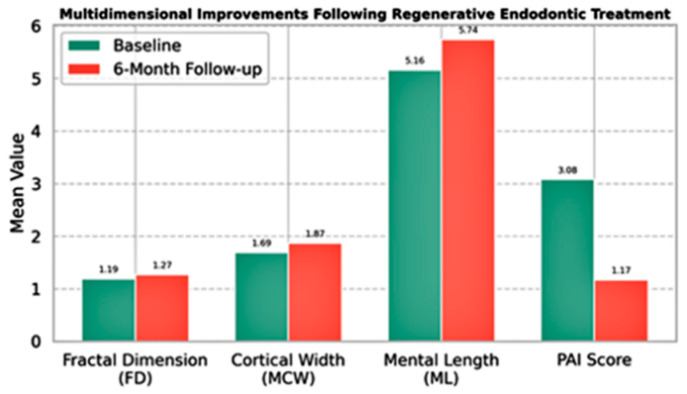
Multidimensional radiographic and morphometric changes observed before and after regenerative endodontic treatment. The bar chart illustrates mean values at baseline and at 6-month follow-up for four key parameters: FD representing trabecular microarchitecture, MCW and ML reflecting macrostructural bone changes, and PAI indicating clinical healing status. Statistically significant improvements were observed in FD, MCW, and ML values, along with a marked reduction in PAI scores. Collectively, these changes suggest a favorable bone response and periapical healing following regenerative therapy.

**Figure 6:**
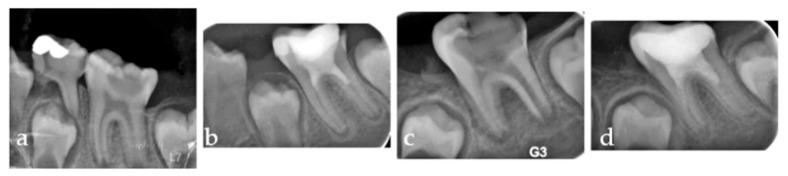
Series of periapical radiographs illustrating two separate clinical cases. (**a**) Pre-operative radiograph of the first case showing a distinct periapical radiolucency corresponding to a PAI score of 4. (**b**) Six-month follow-up of the first case demonstrating significant periapical healing, with the PAI score reduced to 2. (**c**) Pre-operative radiograph of the second case presenting a large periapical lesion, also corresponding to a PAI score of 4. (**d**) Six-month follow-up of the second case showing complete resolution of the lesion, with the periapical area appearing healthy and achieving a PAI score of 1.

Overall, this study’s findings show that regenerative endodontic treatment may improve the complexity of periapical trabecular bone, as seen in higher FD values. The study also found improvements in mandibular bone structure, especially in cortical bone thickness and ML, indicating localized bone remodeling due to treatment. The lack of significant correlations with age or gender suggests these results apply broadly to pediatric populations (Figure 5). Moreover, the significant decrease in PAI scores supports the radiographic evidence of periapical healing. Together, these results demonstrate the effectiveness of regenerative endodontic procedures in not only revitalizing pulpal tissue but also promoting periapical bone regeneration in growing individuals (Figure 6).

## 4. Discussion

RET have recently emerged as a more sustainable, tissue-engineering-based solution for managing pulp necrosis in immature permanent teeth [13]. RET protocols are designed not only to promote continued root development but also to facilitate comprehensive soft and hard tissue healing within the periapical region. Consequently, evaluating the success of this therapeutic approach increasingly requires assessment not solely by the regression of clinical symptoms but also by quantifiable radiological and microstructural changes [14,15]. In this study, we quantitatively assessed the effects of RET on periapical healing and trabecular bone structure in immature permanent teeth, utilizing FD and various panoramic radiomorphometric indices, specifically MCW, PMI, and PAI.

Our findings unequivocally demonstrate that RET not only facilitates pulpal revascularization in immature permanent teeth but also profoundly impacts the structural integrity and organization of periapical trabecular bone tissue. Specifically, the statistically significant increase in FD values, as determined by fractal analysis, provides robust evidence that the regenerative process directly influences bone microarchitecture.

Fractal analysis, by statistically describing the complexity and level of organization of bone trabecular structure, stands out as a reliable quantitative technique for monitoring bone regeneration [9,16]. Rising FD values are generally understood to indirectly indicate increased density, integrity, and organization within the trabecular structure. The utility of fractal analysis in clinical practice is further underscored by its applicability, particularly with traditional two-dimensional radiographs [17]. In our study, the substantial increase in FD values from 1.19 ± 0.07675 at baseline to 1.27 ± 0.05 at the 6-month follow-up (*p* = 0.001) in RET-treated teeth strongly indicates a favorable microstructural response of the periapical bone to regenerative treatment. This aligns with a systematic review by Barros de Oliveira et al. (2025), which reported a correlation between increasing FD values after endodontic treatment and apical bone healing [11]. Similarly, another systematic review published in 2021 noted that fractal analysis is an effective non-invasive method for longitudinal monitoring of bone healing [16]. The absence of a significant change in FD values in the contralateral control group within our study reinforces the notion that the observed increase in the treated group is directly attributable to RET-induced bone remodeling, rather than merely age-related growth.

It is important to contextualize our findings with existing literature. A study by Yu et al. (2009) reported a significant decrease in the FD of reactive bone within 6 months following successful conventional root canal treatment [18]. This finding may seem contradictory but can be explained by the different biological processes at play. This observation from Yu et al. is consistent with the hypothesis that dense reactive bone would normalize towards a more typical density post-treatment. In contrast to Yu et al.’s findings, the observed increase in FD values in the periapical bone microarchitecture following RET in our study suggests an enhancement in the structural complexity and organization of the bone during the healing and remodeling process, rather than a reactive reduction. While both studies employ FD analysis as an indicator of bone healing, the underlying biological processes and dynamic responses differ. Specifically, Yu et al.’s research primarily focused on the normalization of pre-existing reactive bone in the context of periapical lesion resolution, whereas our study assessed bone formation and maturation actively induced by pulpal revascularization in immature permanent teeth. This highlights a key distinction in the mechanisms of bone repair studied. This demonstrates the versatility of fractal analysis as a tool to capture distinct biological responses, whether it is the return of dense reactive bone to a normal state or the active formation of new, organized trabecular bone.

Fractal analysis has indeed been increasingly utilized to monitor bone healing and structural changes across various clinical contexts, including apical periodontitis and endodontic interventions. In alignment with previous studies reporting a significant increase in FD values following successful root canal treatment or retreatment [14,19], our findings also demonstrated a marked enhancement in trabecular complexity post-RET. Specifically, the FD increase from 1.19 to 1.27 observed over a six-month period strongly suggests active bone remodeling and reorganization in the periapical region of immature teeth. This numerical improvement was further corroborated by reductions in PAI scores, underscoring the potential of FD as a valuable, non-invasive marker for assessing the biological success of RET. Given its compatibility with standard two-dimensional radiographs and sensitivity to microstructural changes, FD analysis can serve as a practical and accessible tool for the longitudinal monitoring of bone regeneration processes in clinical endodontics.

Beyond microarchitectural changes identified through fractal analysis, macroscopic and clinical indicators consistently supported the positive outcomes of RET [19,20]. The concurrent improvements observed in both qualitative and quantitative measures across different analytical approaches, including significant increases in morphometric parameters like MCW and ML and favorable reductions in PAI scores, collectively underscore the comprehensive healing capacity of RET. This multi-faceted assessment highlights the importance of integrating various diagnostic tools to gain a complete understanding of the periapical healing process.

Increases in MCW and ML observed in our morphometric analyses can be interpreted as macroscopic reflections of the microstructural improvement occurring in the periapical region. The statistically significant increases in these parameters suggest that a localized bone formation process is activated in the cortical bone thickness and the underlying mandibular skeletal structure following regenerative treatment. As one study indicated, morphological analysis of the mandibular cortex reliably reflects bone density and remodeling processes related to functional loading [20]. De Moraes Ramos et al. further emphasized that MCW measurements can reflect not only age-related bone changes but also specific local treatment effects [21]. The observed increase in ML in our study might similarly be viewed as a morphometric sign of skeletal remodeling. Measurements like MCW and ML demonstrate that changes in bone quality can be identified even on standard two-dimensional radiographs. Additionally, the fact that our study’s PMI values did not significantly alter indicates that the mandible’s general structural proportions were maintained following RET, suggesting that the observed bone growth occurred specifically in localized regions rather than a generalized skeletal change.

Regarding periapical status, we observed that post-treatment PAI scores decreased to levels 1 or 2 the vast majority of cases with available follow-up data that initially presented with pretreatment PAI scores ≥ 3. This finding strongly supports that RET effectively reduces apical inflammation and actively promotes periapical repair. Our results, which showed both decreased PAI scores and increased FD values, align with the findings of Tosun et al., who evaluated the success of endodontic retreatments in both single-session and two-session protocols [22]. Their studies, like ours, demonstrated decreased PAI scores and significant increases in FD values in cases considered cured. Furthermore, our findings are consistent with a randomized clinical trial by Karaoğlan et al., which reported similar success rates between single-session and two-session retreatments as measured by the PAI method [23]. While our results demonstrate a high success rate, it is crucial to contextualize this finding within the broader literature. Our approach, utilizing both the PAI method and quantitative fractal analysis, provides a more objective assessment compared to some past studies that relied on subjective visual evaluations [11,24]. The systematic review we referenced also aligns with our findings of increased FD values post-treatment, supporting the notion of bone formation [11]. However, it highlights that the overall certainty of evidence for this type of analysis is considered “very low” due to methodological limitations in existing studies. This underscores the need for additional, high-quality prospective studies with longer follow-up periods to confirm these promising results and solidify the role of RET in clinical practice.

### 4.1. Clinical Relevance

This study’s findings hold significant clinical relevance. The use of a simple, non-invasive method like fractal analysis on routine panoramic radiographs allows clinicians to objectively monitor and quantify periapical bone healing. The observed increases in FD and mandibular cortical width provide a valuable radiographic indicator of treatment success beyond subjective clinical signs. These results can guide treatment planning and provide a more robust basis for assessing the biological response to regenerative endodontic procedures in children. In summary, this study provides compelling evidence that regenerative endodontic treatments effectively promote favorable periapical bone healing and microarchitectural improvements in immature permanent molars. Our robust quantitative and objective evaluation of microstructural bone changes through fractal analysis, coupled with the integrated assessment of multiple radiomorphometric indices (MCW, ML, and PMI), allowed for a holistic understanding of both cortical and trabecular responses. The distinct improvements observed in FD, MCW, ML, and PAI scores collectively underscore the comprehensive healing capacity of RET. By focusing on young individuals, our findings offer valuable insights into bone healing and potential root development during this crucial growth phase following regenerative therapy. Our results suggest that fractal analysis, combined with radiomorphometric indices, offers a promising approach to inform clinical decision-making and patient care.

### 4.2. Limitations and Future Directions

Although our retrospective approach naturally faced challenges in fully controlling all variables during patient selection, it enabled analysis of a relatively large sample size, which is a key advantage for studying such a rare treatment. However, this study should be viewed as establishing a strong base for future research. While our results showed a high success rate, with significant improvements in both FD and PAI scores, it is also possible that these positive outcomes were partly influenced by our limited sample size and the relatively short six-month follow-up period.

Future studies could improve the accuracy of morphometric assessments by using three-dimensional imaging methods like Cone Beam Computed Tomography (CBCT) to better capture volumetric changes. A thorough discussion of the limitations of using two-dimensional radiographs to evaluate three-dimensional bone structures is necessary. Additionally, investigating longer follow-up periods through prospective clinical trials would be valuable for examining long-term tissue stability and comprehensive root development. These future directions will expand on our findings, offering deeper insights into the long-term success of regenerative endodontics.

## 5. Conclusions

This study conclusively demonstrates that regenerative endodontic therapy effectively promotes not only pulp revascularization but also significant improvements in both the microstructural and macroscopic morphometric characteristics of periapical bone tissue. The synergistic application of fractal analysis and radiomorphometric techniques provides an innovative, quantitative, and objective framework for evaluating periapical bone healing, offering valuable insights for clinical decision-making.

## Figures and Tables

**Table 1:** Comparison of FD values before and after regenerative endodontic treatment and in contralateral teeth.

Group	N	Mean FD ± SD	*p*-Value
Regenerative Tooth (Pre-op)	40	1.19 ± 0.08	
Regenerative Tooth (Post-op)	40	1.27 ± 0.05	0.001 *
Contralateral Tooth (Pre-op)	40	1.25 ± 0.05	
Contralateral Tooth (Post-op)	40	1.29 ± 0.27	0.301

* *p* < 0.05.

**Table 2:** FD values in contralateral teeth are compared before and after regenerative endodontic therapy.

Group	N	Mean FD (Post-op) ± SD
Age 6	2	1.33
Age 7	12	1.29
Age 8	5	1.23
Age 9	9	1.25
Age 10	6	1.27
Age 11	3	1.24
Age 12	1	1.24
Age 13	1	1.34
Age 14	1	1.27
Male	15	1.25 ± 0.05
Female	25	1.28 ± 0.05

**Table 3:** Mandibular morphometric measurements before and after treatment.

Parameter	Side	Time	N	Mean ± SD	*p*-Value
MCW	Left	Before	40	1.68 ± 0.32	
MCW	Left	After	40	1.90 ± 0.33	0.005
MCW	Right	Before	40	1.70 ± 0.272	
MCW	Right	After	40	1.83 ± 0.34	0.049
ML	Left	Before	40	5.10 ± 1.22	
ML	Left	After	40	5.72 ± 1.15	0.022
ML	Right	Before	40	5.23 ± 1.25	
ML	Right	After	40	5.77 ± 1.19	<0.001
PMI	Left	Before	40	0.34 ± 0.07	
PMI	Left	After	40	0.34 ± 0.07	0.990
PMI	Right	Before	40	0.34 ± 0.01	
PMI	Right	After	40	0.32 ± 0.06	0.371

**Table 4:** PAI score distribution and statistical analysis results.

PAI Score	Baseline (n)	Post-Treatment (n)	Description
PAI 1	0	33	Normal periapical structure
PAI 2	3	7	Minor bone changes, not pathognomonic
PAI 3	32	0	Bone structure changes with mineral loss
PAI 4	5	0	Well-defined radiolucency, active periodontitis
**Test**	**Value**	**df**	***p*-value**
Pearson Chi-Square	67.72	3	<0.001
Likelihood Ratio	89.72	3	<0.001
Linear-by-Linear Association	63.03	1	<0.001

## Data Availability

The data presented in this study are not publicly available due to ethical and privacy restrictions. However, anonymized datasets may be made available from the corresponding author upon reasonable request and with appropriate institutional approval.

## Abbreviations

The following abbreviations are used in this manuscript:

FD	Fractal Dimension
ROI	Region of Interest
RET	Regenerative Endodontic Treatment
TIFF	Tagged Image File Format
MCW	Mandibular Cortical Width
MI	Mental Index
CBCT	Cone Beam Computed Tomography
PAI	Periapical Index
PDGF	Platelet-Derived Growth Factor
VEGF	Vascular Endothelial Growth Factor
TGF-β	Transforming Growth Factor-beta
SD	Standard Deviation
SPSS	Statistical Package for the Social Sciences
